# Ecophysiological responses to different forest patch type of two codominant tree seedlings

**DOI:** 10.1002/ece3.1368

**Published:** 2014-12-21

**Authors:** Renyan Duan, Minyi Huang, Xiaoquan Kong, Zhigao Wang, Weiyi Fan

**Affiliations:** 1 College of Life Science, Anqing Normal UniversityAnqing, 246011, China; 2 College of Life Science, Shaanxi Normal UniversityXi'an, 710062, China

**Keywords:** Biomass allocation, coexistence mechanism, ecophysiological response, patch

## Abstract

According to gap-phase dynamics theory, forests can be divided into four distinct patch types: gap patch (G), building patch (B), mature patch (M), and degeneration patch (D). Varying light conditions across patch types are one of the most important factors affecting the coexistence of vegetation. Mechanisms of coexistence can be understood through detailed knowledge of ecophysiological responses of codominant tree seedlings to patch types. The following study was conducted to determine ecophysiological responses of *Cyclobalanopsis glauca* (an evergreen broad-leaved species) and *Bothrocaryum controversum* (a deciduous broad-leaved species) to four different patch types. During the gap-phase dynamics, light intensity and the magnitude of change in the four different patches followed the order of: G > B > D > M. Both species had the greatest photosynthetic capacity in the G patch. Dry leaf mass per area (*LMA*), Chlorophyll a + b concentration (*Chl*), carotenoids (*Car*), and nitrogen content per area (*N*_*a*_) all responded to changes in light across patch type, but *B. controversum* showed greater sensitivity and changes than *C. glauca*. From G to M patch, the maximal quantum efficiency of PSII (*F*_*v*_*/F*_*m*_) had a larger variation magnitude for *B. controversum* than for *C. glauca*. From G to M patch, *B. controversum* showed significant changes in gas exchange, while *C. glauca* showed only small changes. Ecophysiological trait partitioning of response to light in different patches provides a possible explanation of a coexistence mechanism.

## Introduction

Tree species have varying leaf life spans, nitrogen concentrations, respiration rates, carboxylation rates, and other traits that are collectively described species' ecophysiological traits (Kitao et al. 2000; Walters and Reich 2000; Miller et al. 2004; Niinemets 2010; Hitsuma et al. 2012). Ecophysiological traits are considered to influence where species can appear, which species can coexist, and how species respond to light conditions such as shading and full sun (Yoshimura 2010; Wyka et al. 2012). Studies on species distribution and coexistence often demonstrate that some environment factors can induce the plastic response of important ecophysiological traits (Miller et al. 2004; Niinemets 2010; Hitsuma et al. 2012). Vegetation is likely to experience heterogeneous patch habitats throughout the gap-phase dynamics. According to a theory of gap-phase dynamics proposed by Whitmore (1989), cycle progress is initiated by a natural disturbance. Four distinct patches may be recognized: a gap patch (G), building patch (B), mature patch (M), and degeneration patch (D) (Whitmore 1989). The forest community is composed of different patches, and the heterogeneous light conditions in the different patches can lead to changes in patch development and dynamics (Whitmore 1989).

Species-specific responses to heterogeneous environments reflect a number of adjustment mechanisms that enable plants to optimize gas exchange and resource investment strategy. Vegetation foliage modifies its ecophysiological and morphological trait to optimize ambient light utilization as light conditions change (Miller et al. 2004; Niinemets 2010; Hitsuma et al. 2012). Many studies have identified important species-specific light-harvesting factors (e.g., Yoshimura 2010; Wyka et al. 2012), but few have simultaneously researched the suite of other important ecophysiological traits in different patches. Species-specific ecophysiological traits may be very important to their coexistence along G-B-M-D gradients with gap-phase dynamics, but ecophysiological traits responsible for plant functional types are not fully understood.

Leaves of evergreen species are thicker and longer-lived than leaves of deciduous species (Reich et al. 1998). In particular, it is unclear how plants of different functional groups coexist in terms of ecophysiological trait partitioning. *Cyclobalanopsis glauca* and *Bothrocaryum controversum* are common codominant tree species in the evergreen and deciduous broad-leaved forests of subtropical China. We hypothesize that ecophysiological traits of the two codominant species response to light environments along gap-phase dynamics (G-B-M-D) are similar, but that *B. controversum* (a deciduous species) has higher plasticity than *C. glauca* (an evergreen species) during the gap-phase dynamics, because deciduous plants are more sensitive to light changes than evergreen plants (Cao 2001; Miyazawa and Kikuzawa 2006; Böhnke and Bruelheide 2013). Here, we used ecophysiological traits of *C. glauca* and *B. controversum* for identifying their niche partitioning within the gap-phase dynamics.

## Materials and Methods

### Study site and species

An evergreen and deciduous broadleaf mixed forest was selected on the southern slope of the Dabieshan Mountain (31°02′35′′ N, 116°06′47′′ E, and 680 m a.s.l) in Anhui, eastern China. According to data from Yaoluoping climate station (30°59′07′′ N, 116°04′′56′′ E, 1060 m a.s.l.), the mean annual air temperature is 12.3°C, and the mean annual precipitation is 2032.6 mm, falling mainly in the summer (44.2%). The codominant tree species in the experimental site (100 × 100 m) are *B. controversum* and *C. glauca*. The stand age is more than 150 years old by the tree ring analysis of the highest tree. The main subdominant tree species include evergreen plants, such as *Ilex pedunculosa*, *Eurya muricata,* and *Cyclobalanopsis gracilis*, and deciduous plants, such as *Rhododendron simsii, Platycarya strobilacea,* and *Lindera glauca*. Common herbs are *Cardamine flexuosa*, *Salvia farinacea, Arthraxon hispidus,* and *Polygonum bungeanum*, while common vines included *Ficus sarmentosa* and *Paederia scandens*.

*Cyclobalanopsis glauca* and *B. controversum* seedlings were raised from seeds in the greenhouse of Anqing Normal University. All seeds of the same species were collected from the same tree in autumn 2009. In spring 2010, seeds were sown in 30 × 40 cm trays filled with a mixed substrate of sand and potting soil (1:2, v/v). All seeds were exposed to a 24/19°C day/night cycle and 14 h daytime. Three weeks after germination, they were transplanted to pots (1.6 L) filled with the same substrate. Plants were weeded periodically and watered to saturation to avoid water stress. In April 2010, 60 seedlings per species were selected and transplanted into 3.0-L pots filled with a mixed substrate of compost, vermiculite, and top forest soil from our field site (1:1:3, v/v/v).

### Patch chosen

**Figure 1:**
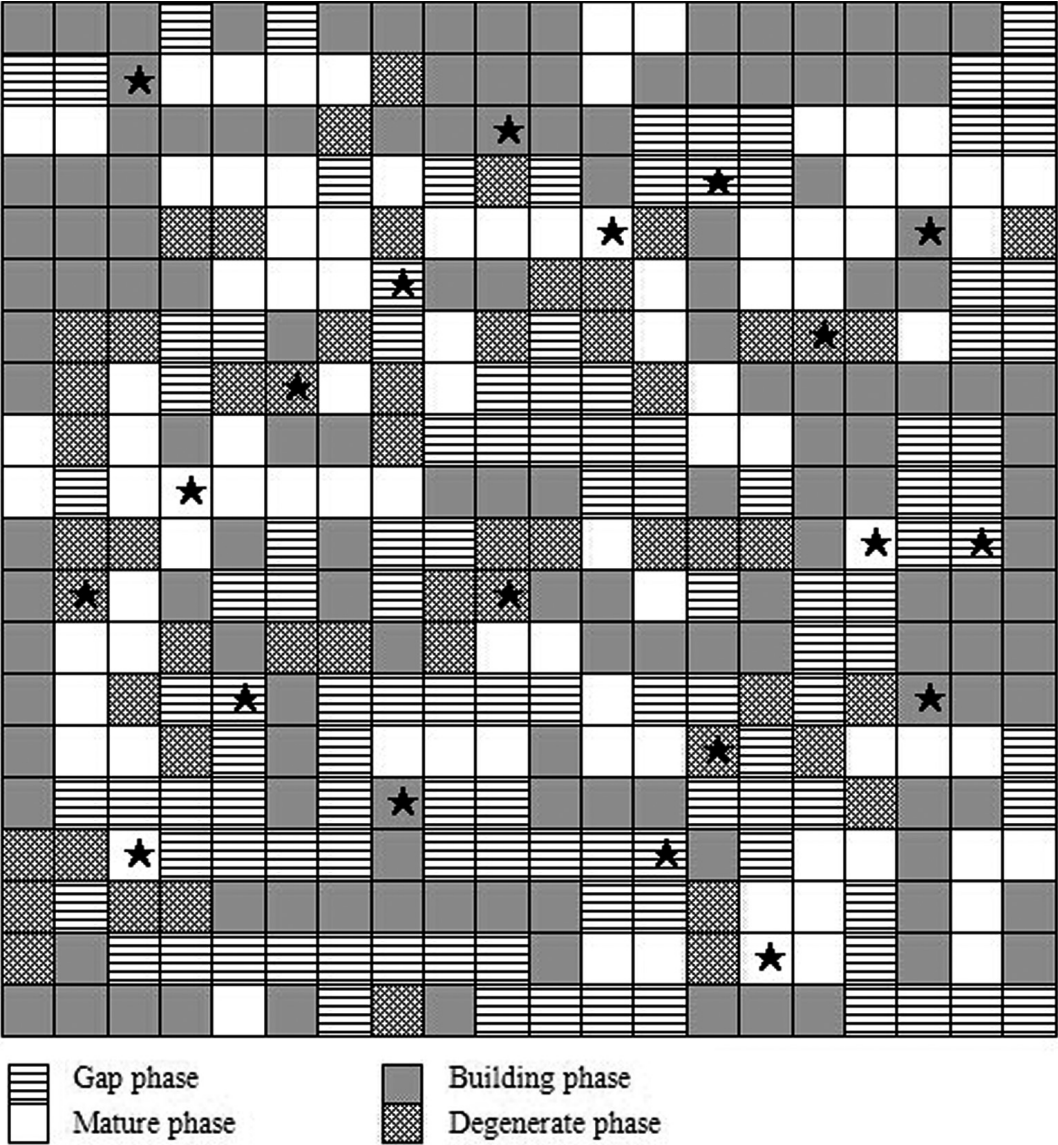
The mosaics patch in an evergreen and deciduous broadleaf mixed forest (100 m × 100 m). The 20 patches with blue box are the selected patches.

The experimental site was divided into 400 quadrats (5 × 5 m) (see Fig.1). Height and *DBH* (diameter at breast height (cm)) of all trees were measured. Average tree height (*H* (m)) and the average height of the canopy trees (*HC*) were 6.9 m and 10.2 m, respectively. Using Daubenmire's cover class method (Daubenmire 1959), we measured the percent cover of each quadrat (5 × 5 m).

Combining our observations and field work, we identified the following four development patches (Table1). Gap patch (G): an open area with the brightest light conditions in the experimental site and identifiable gap makers and/or decomposing dead logs. The average tree height of regenerating layer is less than half of that of canopy layer. Building patch (B): average tree height exceeds half of the average tree height of canopy layer. Some trees are close to the height of the main canopy layer. Mature patch (M): consists of mainly canopy trees with heights greater than the average tree height of the surrounding canopy. Degenerate patch (D): some large emergent trees with hollow trunks, sparse foliage, bald necrotic holes, and/or spots in the stems. The height of emergent trees is the same or exceeds the height of main canopy layer.

**Table 1:** The characteristics and classification standards of mosaics patch in an evergreen and deciduous broadleaf mixed forest

Patches	Characteristics			Classification standards					
	*DBH*_*q*_ (cm)	*H*_*q*_ (m)	*HC*_q_ (m)	*H*_*q*_ (m)	*HC*_*q*_ (m)	*H*_max_ (m)	Gap Makers	Emergent trees	Coverage (%)
G	4.01 ± 0.34	1.01 ± 0.47	2.13 ± 0.62	<6.9	<6.9	<6.9	Yes	No	0–10
B	8.03 ± 2.12	4.74 ± 1.56	7.64 ± 3.11	<6.9	6.9∼10.2	<10.2	No	No	10–30
M	16.27 ± 2.98	10.65 ± 3.12	13.61 ± 4.34	>6.9	>10.2	>10.2	No	No	60–100
D	14.32 ± 2.45	8.12 ± 2.45	18.64 ± 5.17	>6.9	>10.2	>10.2	No	Yes	40–60

Data are presented as mean ± standard error (SE). *DBH*_*q*_: average DBH. *H*_max_: maximum tree height. *H*_*q*_: average tree height in the quadrate (5 m × 5 m). *HC*_*q*_: average tree height of canopy layer in the quadrate (5 m × 5 m). Emergent trees: tree height exceeds the main canopy layer in the sample. They have sparse leaves, broken branches, and their stems have some bald necrotic spots or holes.

### Experimental design

In March 2011, *C. glauca* and *B. controversum* seedlings that had been fostered in a greenhouse were transplanted to 20 randomly selected natural patch types (five replicates of G, M, B, and D patches). There were three *C. glauca* seedlings and three *B. controversum* seedlings in each patch. Each seedling was planted at random. Some herb and vine plants grew around our studied seedlings. Especially, parts of the stems and leaves may grow toward or twine around the trunk of the seedlings of *C. glauca* and/or *B. controversum*. To avoid their influence on the growth and survival of the seedlings, we removed the herb and vine plants around our studied seedlings. Throughout the experiment, seedlings were watered to saturation more than twice weekly to avoid water stress.

### Light intensity

To reflect daily variation of light intensity in different patches, we used a scout™ light meter (3415FSE Dual Solar/Electric Quantum Meter). To compare the difference of light in four patch types, we randomly selected one patch type in 20 patches to measure the light intensity of every patch at a height of 1.60 m above the ground. This standard height was chosen as seedling height varied across the patch types. The light intensity in all four selected patches was measured every 2 h from 6:00 to 18:00 on each cloudless day (5–11 July 2012). The 20 values of light intensity in less than 1 min were recorded exactly at the same time by four researchers (one researcher in a patch) in each patch type.

### Gas exchange measurement

Leaf gas exchanges were measured from 8:30 to 10:30 on sunny days (July 12–22, 2012) in the mid-to-upper parts of leaves (the region of the individual crown) of the selected plants. Only current year, fully developed leaves were selected to reduce the possible effect of leaf age. We used a portable photosynthesis measurement system (LI-6400, LI-COR Inc. Lincoln, NE) to determine the light and CO_2_ photosynthetic response curves.

Light response curves for both species were generated using 12 photosynthetic photon flux density (PPFD: 2000, 1500, 1000, 800, 600, 400, 200, 100, 50, 25, 15, and 0 *μ*mol photons m^−2^s^−1^) with a stable CO_2_ concentration (400 *μ*mol·mol^−1^). All measurements were obtained at a leaf temperature of 25°C, and the leaf-to-air vapor pressure difference (LVPD) was < 1.0 kPa. The maximal net photosynthetic rate (*P*_nmax_) and the dark respiration rate (*R*_d_) were derived from the light response curves using Photosyn Assistant Ver. 1.1.2 software (Dundee Scientific, Dundee, UK). At their saturating PPFD, CO_2_ photosynthetic response curves were measured with similar leaf temperature (25°C) and LVPD (< 1.0 kPa). The leaves were exposed to 400 *μ*mol mol^−1^ CO_2_ and 1000 *μ*mol·m^−2^·sec^−1^ PPFD until their gas exchange was steady (at least 15 min). After steady-state photosynthesis was induced, we measured the responses of the photosynthetic rate (*P*_n_) to changes in intercellular CO_2_ concentration (*C*_i_). The CO_2_ concentration (*C*_*a*_) was set to 400 *μ*mol·mol^−1^, and then it was sequentially lowered to 300, 200, 150, 100, and 50 *μ*mol mol^−1^. Following this, *C*_*a*_ was returned to 400 *μ*mol·mol^−1^, and then we measured CO_2_ assimilation rate at four levels *C*_*a*_: 800, 1000, 1200, and 1500 *μ*mol·mol^−1^. The *P*_n_–*C*_i_ curves were constructed from the measured values of *P*_n_ and *C*_i_ at each given *C*_*a*_. The electron transport used in regeneration of RuBP (*J*_max_), the maximal rates of carboxylation by Rubisco (*V*_cmax_), and photosynthetic energy transformation efficiency (*δ*) were calculated as described by Farquhar and Sharkey (1982), Loustau et al. (1999) and Schreiber et al. (1986), respectively.

### Chlorophyll fluorescence measurement

After gas exchange measurements, we estimated the chlorophyll fluorescence parameters on the same leaves used for the gas exchange measurements using a portable pulse-modulated fluorometer (PAM-2100; Walz, Effeltrich, Germany) connected to a computer running control software. One *B. controversum* seedling and one *C. glauca* seedling were randomly selected in each patch. Total eight seedlings (four *B. controversum* seedlings and four *C. glauca* seedlings) were measured in four selected patches (the same four patches as those measured in light intensity).

For consecutive measurements of chlorophyll fluorescence parameters, we marked a fixed position on each sampled leaf. To measure the minimal fluorescence (*F*_0_) and maximal fluorescence (*F*_m_) parameters, the sample was first dark-adapted for at least 20 min using a leaf clip. The maximal photochemical efficiency of PSII (*F*_v_*/F*_m_ = (*F*_m_ − *F*_0_)/*F*_m_) was calculated. The photochemical quenching (*qP*), nonphotochemical quenching (*qN*), actual photochemical efficiency of PSII in the light (*ΦPSII*), and apparent electron transport rate (*ETR*) were measured at 2-h intervals from 6:00 to 18:00 on 23–29 July 2012.

### Leaf morphology

Following the measurements of chlorophyll fluorescence and gas exchange, we harvested the leaves, extracted leaf disks (1 cm diameter), and then separated these leaf disks into two parts. One part was used to determinate the chlorophyll a (*Chla*), chlorophyll b (*Chlb*), carotenoid (*Car*), and total chlorophyll content per unit area (*Chl*) using UV-vis spectrophotometry after extraction with dimethylformamide. The other part was dried at 60°C for 48 h. We calculated the *LMA* according to the dry weight per area and then ground these dried leaves to fine powder for nitrogen analysis by the Kjeldahl method. Finally, we calculated photosynthetic nitrogen use efficiency (*PNUE* = *P*_*n*max_/*N*_a_), the ratio of *Chl* to *N*_*a*_ (*Chl*_N_), and the ratio of *Chla* to b (*Chla/b*), where *N*_a_ is the area-based leaf nitrogen content. According to the method proposed by Niinemets and Tenhunen (1997), we assessed the leaf nitrogen allocated to bioenergetics (*P*_B_), Rubisco (*P*_R_), and light-harvesting components (*P*_L_).

### Seedling survival, height growth, and dry weight

When the experiment ended (July 2012), seedling survival rates per species and patch were recorded. Seedling height was measured, and relative growth rate (RGR) was calculated as *RGR* = (*ln w*_2_ − *ln w*_1_)/(*t*_2_ − *t*_1_), where *w*_2_ and *w*_1_ represent the final and initial height at time *t*_2_ and *t*_1_, respectively. Four seedlings per species and treatment (*n* = 32) were harvested, and they were washed carefully to avoid losing fine roots. They were oven-dried at 70°C for 72 h, and the dry weight of roots, shoots, and leaves was measured. The root mass ratio (*RMR*), shoot mass ratio (*SMR*), and leaf mass ratio (*LMR*) were calculated as ratio of dry weight of each fraction to the total biomass.

### Statistical analysis

Statistica v8.0 (StatSoft Inc. Tulsa, Oklahoma, USA) was used to perform all analyses. Each value of ecophysiological characteristics was presented as the mean and standard error (SE).

Spatial autocorrelation of the selected patched was calculated as Moran's I (Fortin and Dale 2005), and we did not observe that there lied a spatial autocorrelation. The survival of seedlings was analyzed with the log-likelihood ratio test (G-test). Differences between species and treatments were analyzed by repeated measures ANOVA using Tukey's test. Before ANOVA, data were log-transformed to meet assumptions of homogeneity of variance and normality when necessary.

## Results

### Light intensity

**Figure 2:**
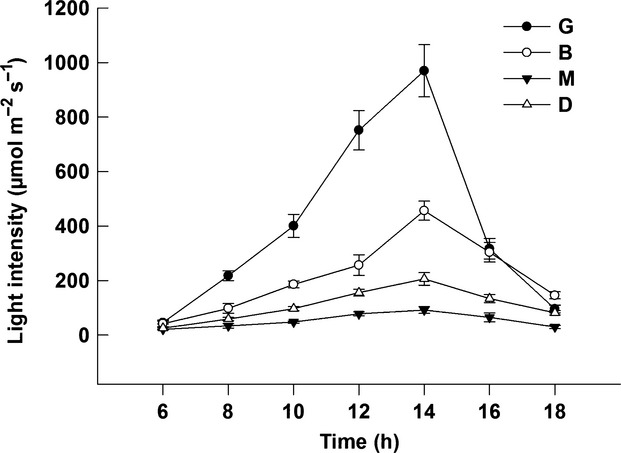
The diurnal variation of the light intensity in four selected patches in the gap-phase dynamics (G, B, M and D) at a height of 1.60 m on seven cloudless days (5–11 July 2012).

From 06:00 h to 18:00 h, light intensity followed a diurnal pattern increasing to midday and then decreasing. The greatest value appeared at 14:00 h (Fig.2). Light intensity and the change magnitude (the difference of the max value and the min value from 6:00 to 18:00) in the four different patches followed the order of: G > B > D > M (Fig.2).

### Photosynthetic traits

For *B. controversum*, the maximum net photosynthetic rate (*P*_nmax_), the photosynthetic nitrogen use efficiency (*PNUE*), the photosynthetic energy transformation efficiency (*δ*), the maximum carboxylation rate (*V*_cmax_), and the maximum electronic transfer rate (*J*_max_) differed significantly among the four different patches. The highest values were in the G patch, and the lowest values were in the M patch (Table2). All measured gas exchange parameters for *C. glauca* were not significantly different among four patches. The dark respiration rate (*R*_d_) for *C. glauca* was significantly different (Table2). In the G patch, the gas exchange parameters (*P*_nmax_, *PNUE*, *δ*, *V*_cmax_, and *J*_max_) of *B. controversum* were higher than those of *C. glauca* (Table2), but in the M patch, these parameters of *B. controversum* were lower than those of *C. glauca* (Table2). Compared to the other three patches, *B. controversum* and *C. glauca* in the M patch both had the lowest values of the leaf nitrogen allocated to Rubisco (*P*_R_) and bioenergetics (*P*_B_), as well as the highest leaf nitrogen allocated to light-harvesting components (*P*_L_) (Table2). Only *P*_L_ showed a significant difference among the four patches for both tree species.

**Table 2:** The leaf gas exchange parameters in *Bothrocaryum controversum* and *Cyclobalanopsis glauca* seedlings in the gap phase dynamics (G, B, M, and D)

Species	Patches	*P*_nmax_	*R*_d_	*PNUE*	*δ*	*V*_cmax_	*J*_max_	*P*_R_	*P*_B_	*P*_L_
*B. controversum*	G	10.15 ± 1.89 a	0.26 ± 0.03 a	11.25 ± 1.53 a	0.23 ± 0.03 a	33.02 ± 4.68 a	70.13 ± 6.23 a	0.28 ± 0.09 a	0.07 ± 0.01 a	0.12 ± 0.05 c
	B	6.54 ± 0.95 b	0.25 ± 0.03 a	10.73 ± 2.09 ab	0.18 ± 0.05 ab	30.76 ± 3.36 a	62.57 ± 5.27 a	0.25 ± 0.06 a	0.06 ± 0.01 a	0.19 ± 0.04 b
	M	3.27 ± 0.39 c	0.21 ± 0.01 a	6.96 ± 1.12 b	0.10 ± 0.04 b	18.78 ± 2.14 b	36.41 ± 4.52 b	0.23 ± 0.05 a	0.05 ± 0.01 a	0.29 ± 0.03 a
	D	8.11 ± 0.07 ab	0.26 ± 0.03 a	10.98 ± 2.16 ab	0.17 ± 0.05 ab	28.58 ± 4.24 a	53.67 ± 4.69 a	0.26 ± 0.05 a	0.05 ± 0.01 a	0.18 ± 0.04 b
*C. glauca*	G	4.68 ± 0.86 a	0.24 ± 0.01 a	7.05 ± 0.89 a	0.18 ± 0.02 a	30.98 ± 4.68 a	59.17 ± 8.46 a	0.34 ± 0.06 a	0.08 ± 0.01 a	0.15 ± 0.03 b
	B	4.35 ± 0.39 a	0.16 ± 0.02 b	7.44 ± 1.23 a	0.16 ± 0.02 a	27.91 ± 6.22 a	57.24 ± 9.22 a	0.32 ± 0.05 a	0.07 ± 0.01 a	0.21 ± 0.04 ab
	M	4.11 ± 0.78 a	0.08 ± 0.02 c	7.88 ± 0.96 a	0.14 ± 0.03 a	26.46 ± 4.18 a	50.09 ± 6.98 a	0.30 ± 0.08 a	0.06 ± 0.01 a	0.27 ± 0.05 a
	D	4.41 ± 0.51 a	0.14 ± 0.03 b	7.49 ± 0.87 a	0.16 ± 0.01 a	28.02 ± 4.22 a	55.37 ± 7.64 a	0.31 ± 0.05 a	0.06 ± 0.01 a	0.23 ± 0.02 ab

Data are presented as mean ± standard error (SE). Means with different letters differ significantly in same species and among different patches (a > b > c > d). *P*_nmax_ is the maximum net photosynthetic rate (*μ*mol·m^−2^·sec^−1^), *R*_*d*_ is the dark respiration rate (*μ*mol·m^−2^·sec^−1^), *PNUE* is the photosynthetic nitrogen use efficiency (*μ*mol·g^−1^·sec^−1^), *δ* is the photosynthetic energy transformation efficiency (electrons quanta^−1^), *V*_cmax_ is the maximum carboxylation rate (*μ*mol·m^−2^·sec^−1^), *J*_max_ is the maximum electronic transfer rate (*μ*mol·m^−2^·sec^−1^), *P*_R_, *P*_B_, and *P*_L_ are the leaf nitrogen allocated to Rubisco, bioenergetics, and light-harvesting components, respectively.

### Chlorophyll fluorescence measurements

**Figure 3:**
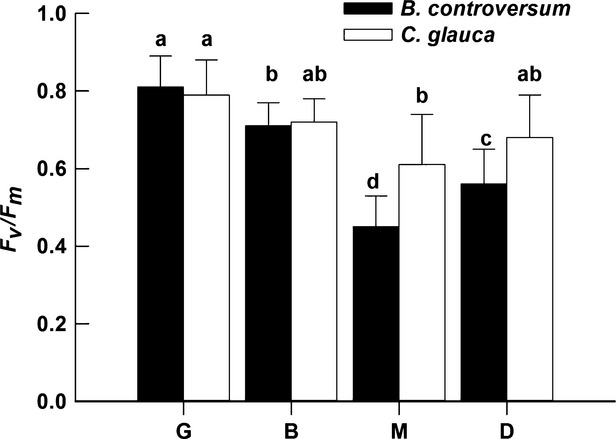
The maximum quantum efficiency of PSII (*F*_v_/*F*_m_) in *Bothrocaryum controversum* and *Cyclobalanopsis glauca* seedlings in the gap-phase dynamics (G, B, M, and D). One *B. controversum* seedling and one *C. glauca* seedling were randomly selected in each patch, except one M patch where all *B. controversum* seedlings died. Total 39 seedlings (19 *B. controversum* seedlings and 20 *C. glauca* seedlings) were measured in all 20 patches. Means with different letters differ significantly in same species and among different patches (a > b > c > d).

**Figure 4:**
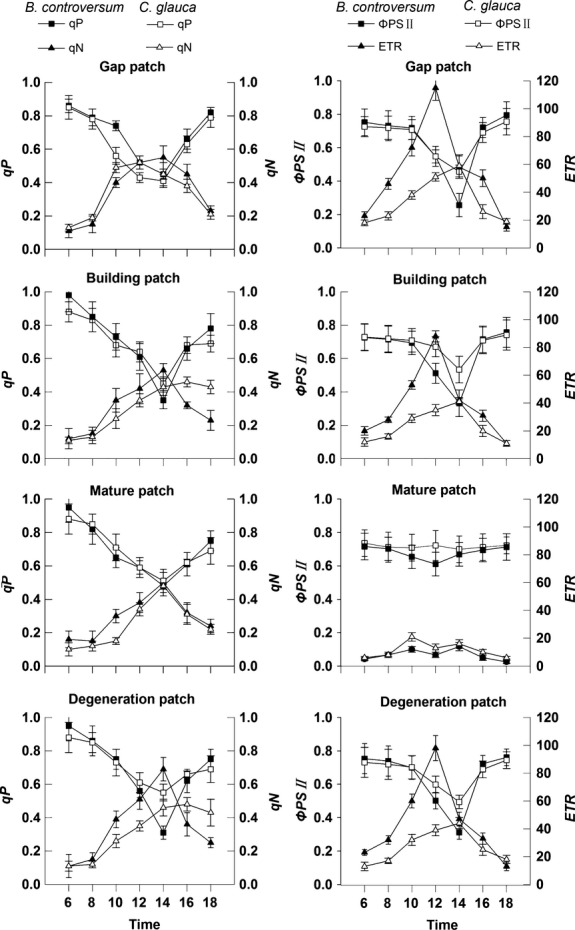
The diurnal variation of chlorophyll fluorescence parameters (*qP*, *qN*, *ΦPSII,* and *ETR*) in *Bothrocaryum controversum* and *Cyclobalanopsis glauca* seedlings in the gap-phase dynamics (G, B, M, and D) on 23–29 July 2012. One *B. controversum* seedling and one *C. glauca* seedling were randomly selected in each patch. Total eight seedlings (four *B. controversum* seedlings and four *C. glauca* seedlings) were measured in four selected patches (the same four patches as those in Fig.2).

In the G patch, the maximal quantum efficiency of PSII (*F*_*v*_*/F*_*m*_) values of *B. controversum* and *C. glauca* was the highest (around 0.8). The two species both had significant differences among the four different patches in the PSII (*F*_*v*_*/F*_*m*_) (Fig.3). From G to M patch, the *F*_v_/*F*_m_ values of *B. controversum* and *C. glauca* decreased by approximately 44.4% and 22.8%, respectively (Fig.3). At G patch, the values of the photochemical quenching (*qP*), the actual photochemical efficiency of PSII (*ΦPSII*), and electron transport rate (*ETR*) measured late morning/early afternoon were higher for *B controversum* than for *C. glauca* (Fig.4). At 14:00 h in the G patch, there was some photoinhibition (a decreasing photosynthetic efficiency with high light at noon) for the two species, as nonphotochemical quenching (*qN*) was more than 0.6, and *qP*, *ΦPSII,* and *ETR* were less than 0.5, 0.4, and 60 *μ*mol electrons m^−2^·sec^−1^, respectively (Fig.4). In the G patch, *qN*, *ΦPSII,* and *ETR* have a greater diurnal variation (the difference of the max value and the min value from 6:00 to 18:00) for *B. controversum* than for *C. glauca* (0.44 vs. 0.39 of *qN*, 0.539 vs. 0.301 of *ΦPSII*, 92 vs. 41 of *ETR*), while *qP* has no significant difference (Fig.4). In the M patch, *qP* and *ΦPSII* have a greater diurnal variation for *B. controversum* than for *C. glauca* (0.47 vs. 0.37 of *qP*, 0.105 vs. 0.037 of *ΦPSII*), while *qN* and *ETR* have no significant difference (Fig.4).

### Leaf morphology

**Figure 5:**
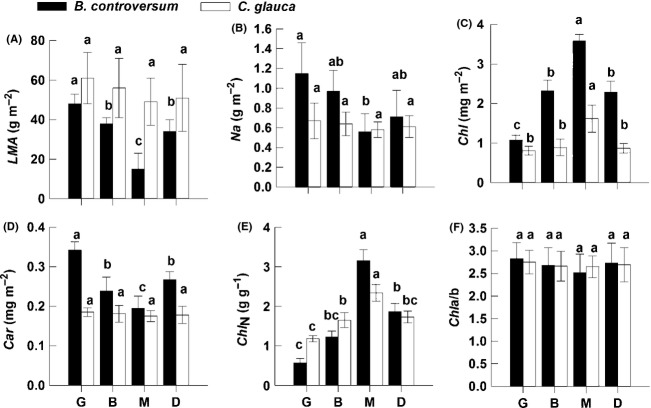
The leaf morphology, pigment, and nitrogen contents in *Bothrocaryum controversum* and *Cyclobalanopsis glauca* seedlings in the gap-phase dynamics (G, B, M, and D). Total 39 seedlings (19 *B. controversum* seedlings and 20 *C. glauca* seedlings) were measured in all 20 patches. The 39 seedlings are same as those in Fig.3. Means with different letters differ significantly in same species and among different patches (a > b > c > d). *LMA* is the leaf dry mass per area (g·m^−2^), *N*_*a*_ is the nitrogen content per area (g·m^−2^), *Chl* is the chlorophyll content per area (mg·m^−2^), *Car* is the carotenoid content per area (mg·m^−2^), *Chl*_N_ is the ratio of *Chl* to *N*_*a*_ (mg·g^−1^) and *Chla/b* is the ratio of *Chla* to b.

*Bothrocaryum controversum* had a lower leaf dry mass per area (*LMA*), but higher nitrogen content per area (*N*_*a*_), Chl a + b concentration per area (*Chl*), and carotenoid (*Car*) in all patches compared to *C. glauca* (Fig.5A–D). *Bothrocaryum controversum* had a significantly lower *LMA*, *N*_*a*_, and *Car* in the M patch compared to the other three patches, while no significant difference was observed for *C. glauca* in all patches (Fig.5A, B, and D). The two species both had significantly higher *Chl* and *Chl*_N_ in the M patch than in the other three patches (Fig.5C,E), and no significant differences in *Chla/b* in all patches (Fig.5F).

### Seedling survival and growth

The survival of *B. controversum* showed a significant change from G to M (92% to 36%), while *C. glauca* showed only a small change (86–70%). Relative growth rate (*RGR*) differed significantly between patches for both species with the greatest values appearing in the G patch and the smallest values in the M patch (Table3). For *B. controversum*, root mass ratio (*RMR*) and shoot mass ratio (*SMR*) decreased significantly and leaf mass ratio (*LMR*) increased significantly from the G to M patch as shading increased (Table3). Conversely, *RMR* and *LMR* for *C. glauca* for the same conditions showed the opposite changes though changes were small and not significant (Table3).

**Table 3:** Survival (%), relative growth rate (RGR, mm·year^−1^), the mass ratios of root (RMR), stems (SMR), and leaf (LMR) for *Bothrocaryum controversum* and *Cyclobalanopsis glauca* seedlings in the gap-phase dynamics (G, B, M, and D)

Species	Patches	Survival	RGR	RMR	SMR	LMR
*B. controversum*	G	92 ± 9 a	0.59 ± 0.11 a	0.42 ± 0.05 a	0.35 ± 0.06 a	0.23 ± 0.06 c
	B	71 ± 8 b	0.41 ± 0.06 b	0.26 ± 0.03 b	0.30 ± 0.07 ab	0.44 ± 0.03 b
	M	36 ± 5 c	0.19 ± 0.04 c	0.13 ± 0.03 c	0.21 ± 0.03 b	0.66 ± 0.06 a
	D	73 ± 7 b	0.45 ± 0.08 b	0.29 ± 0.04 b	0.23 ± 0.03 b	0.48 ± 0.07 ab
*C. glauca*	G	86 ± 16 a	0.38 ± 0.09 a	0.17 ± 0.03 b	0.49 ± 0.07 a	0.34 ± 0.05 a
	B	76 ± 15 ab	0.31 ± 0.06 ab	0.29 ± 0.06 ab	0.40 ± 0.08 ab	0.31 ± 0.07 a
	M	70 ± 10 b	0.28 ± 0.05 b	0.38 ± 0.05 a	0.32 ± 0.06 b	0.30 ± 0.06 a
	D	78 ± 11 ab	0.33 ± 0.06 ab	0.30 ± 0.08 ab	0.44 ± 0.05 ab	0.26 ± 0.07 a

Data are presented as mean ± standard error (SE). Means with different letters differ significantly in same species and among different patches (a > b > c > d).

## Discussion

Light is very important for the survival, growth, and development of higher plants (Valladares et al. 1997; Walters and Reich 2000; Durand and Goldstein 2001; Hitsuma et al. 2012). During the gap-phase dynamics, the light environment varies diurnally and by patch type. Individual plants are in reality fine-tuning the expression of physiological traits (within the range possible given their genetic makeup) to optimize their carbon gain at whatever environmental condition they are experiencing. The leaf is a good representation of the ability of a plant to respond to light changes (Poorter and Bongers 2006).

### Ecophysiological responses to G patch (high light)

Gap is an important forest growth stage driving productivity and plant regeneration (Houter and Pons 2005; Kitaoka et al. 2009; Kuptz et al. 2010). In the G patch, *B. controversum* and *C. glauca* seedlings both had the highest photosynthetic capacity growth ratio, and survival, indicating that a forest gap is beneficial to growth and regeneration. Our results are in agreement with previous gap dynamics research (Gómez-Aparicio et al. 2006; Oguchi et al. 2008; Kitaoka et al. 2009; Kuptz et al. 2010). Photoinhibition was observed midday in the G patch due to high-intensity light conditions. Seedlings need to develop protective mechanisms to reduce light absorbance and avoid photooxidation in high light. These mechanisms include increasing the carotenoid concentration and nitrogen content per area (Gómez-Aparicio et al. 2006), decreasing the ratio of *Chl* to *N*_*a*_, and the chlorophyll content per area. In addition, to prevent high-intensity light damage, species tend to invest more nitrogen in protecting leaf structure (e.g., the epidermis) and invest little nitrogen in photosynthetic organs (Oguchi et al. 2008; Kitaoka et al. 2009; Kuptz et al. 2010). These protective mechanisms were observed in the two codominant tree seedlings in this study.

### Ecophysiological responses to M patch (low light)

Plants with different functional types develop acclimation mechanisms to optimize ambient light utilization under low light conditions (Miller et al. 2004; Yoshimura 2010; Hitsuma et al. 2012; Wyka et al. 2012). Natural growth and regeneration in lower light conditions are related to the photosynthetic capacity in combination with morphological and physiological changes (Gommers et al. 2013). Changes induced by competition for light reflect plant shade avoidance or shade-tolerance ability (Gommers et al. 2013). Under low light in the M patch, shade-avoidance species, like *B. controversum*, invested relatively higher mass ratios to leaf (higher *LMR*) at the expense of root biomass (lower *RMR*), while shade-tolerance species, like *C. glauca*, showed little change. In addition, plants have a trade-off in leaf N allocation to Rubisco, bioenergetics, and light-harvesting components. Higher leaf N allocated to light-harvesting organs under low light is associated with lower investment in Rubisco, which induces lower *PNUE* (Xiang et al. 2013). In our study, the evergreen species, *C. glauca*, showed increased Chl:N ratios, *P*_*L*_, and *PNUE* in the M patch as a result of the increase in chlorophyll, which indicates that the *C. glauca* performs well under low light. *Bothrocaryum controversum* had a poor shade acclimation, indicating a trade-off between high light (in the G patch) and shade adaptation (in the M patch). Different responses in morphology and physiology to low light can induce mortality difference (Gómez-Aparicio et al. 2006; Feijó et al. 2009; Gommers et al. 2013). We observed that the mortality of *B. controversum* was approximately two times higher than that of *C. glauca* in the M patch. The different mortality is a good indicator of their interspecific differences in low light tolerance.

### Heterogeneous ecophysiological trait partitioning to gap-phase dynamics

Environmental resource segregation and trade-offs in ecophysiological traits are commonly used to study whether trait partitioning is beneficial to species coexistence for different functional groups (Gómez-Aparicio et al. 2006; Niinemets 2010). Codominant species have different resource acquisition methods by ecophysiological trait partitioning (Gómez-Aparicio et al. 2006). During the gap-phase dynamics, *B. controversum* exhibited higher degree of change, which was in accordance with its shorter leaf life span and higher potential photosynthetic rates. The greater changes within the gap-phase dynamics were inherently associated with the higher flexibility in utilizing available resources in different patches. *C. glauca* exhibited small changes with slow growth and little variation in ecophysiological traits during the gap-phase dynamics, for evergreen species have a stable physiological performance due to its sclerophyllous and long-living leaves (Böhnke and Bruelheide 2013). The various interspecific responses to the four different patches provide new insights into the coexistence mechanism for *B. controversum* and *C. glauca* with the gap-phase dynamics.

Light interception and utilization efficiency along light gradients changes for plants of different functional groups (Niinemets 2010). A suite of traits, such as the biomass allocation to leaves, leaf chlorophyll content, leaf dry mass per unit area, and leaf nitrogen allocation, at various scales and plasticity determine plant light-harvesting efficiency (Feijó et al. 2009; Niinemets 2010). Mechanisms of light interception by seedlings of different functional groups have been addressed frequently as one of the most important causes of species coexistence and biodiversity conservation in tropical and subtropical forests (Feijó et al. 2009; Niinemets 2010; Gommers et al. 2013). Our results indicate the importance of temporal and spatial variation of light during the gap-phase dynamics and fluctuating ecophysiological trait partitioning for stable coexistence of *B. controversum* and *C. glauca*.

## References

[b1] Böhnke M, Bruelheide H (2013). How do evergreen and deciduous species respond to shade? Tolerance and plasticity of subtropical tree and shrub species of South-East China. Environ. Exp. Bot.

[b2] Cao KF (2001). Morphology and growth of deciduous and evergreen broad-leaved saplings under different light conditions in a Chinese beech forest with dense bamboo undergrowth. Ecol. Res.

[b3] Daubenmire RA (1959). Canopy-coverage method of vegetational analysis. Northwest Sci.

[b4] Durand LZ, Goldstein G (2001). Photosynthesis, photoinhibition, and nitrogen use efficiency in native and invasive tree ferns in Hawaii. Oecologia.

[b5] Farquhar GD, Sharkey TD (1982). Stomatal conductance and photosynthesis. Annu. Rev. Plant Physiol.

[b6] Feijó NSA, Mielke MS, Gomes FP, Franca S, Lavinsky AO (2009). Growth and photosynthetic responses of *Gallesia integrifolia* (Spreng.) Harms and *Schinus terebinthifolius* Raddi seedlings in dense shade. Agroforest Syst.

[b7] Fortin MJ, Dale MRT (2005). Spatial analysis—a guide for ecologists.

[b8] Gómez-Aparicio L, Valladares F, Zamora R (2006). Differential light responses of Mediterranean tree saplings: linking ecophysiology with regeneration niche in four co-occurring species. Tree Physiol.

[b9] Gommers CM, Visser EJ, Onge KRS, Voesenek LA, Pierik R (2013). Shade tolerance: when growing tall is not an option. Trends Plant Sci.

[b10] Hitsuma G, Han QM, Chiba Y (2012). Photosynthesis and growth of *Thujopsis dolabrata* var. *hondai* seedlings in the understory of trees with various phenologies. J. For. Res.

[b11] Houter NC, Pons TL (2005). Gap size effects on photoinhibition in understorey saplings in tropical rainforest. Plant Ecol.

[b12] Kitao M, Lei TT, Koike T, Tobita H, Maruyama Y (2000). Susceptibility to photoinhibition of three deciduous broad leaf tree species with different successional traits raised under various light regimes. Plant, Cell Environ.

[b13] Kitaoka S, Watanabe M, Watanabe Y, Kayama M, Nomura M, Sasa K (2009). Growth of regenerated tree seedlings associated with microclimatic change in a mature larch plantation after harvesting. Landsc. Ecol. Eng.

[b14] Kuptz D, Grams TEE, Günter S (2010). Light acclimation of four native tree species in felling gaps within a tropical mountain rainforest. Trees.

[b15] Loustau D, Beahim M, Gaudillere JP, Dreyer E (1999). Photo-synthetic responses to phosphorous nutrition in two-year-old maritime pine seedlings. Tree Physiol.

[b16] Miller RE, Gleadow RM, Woodrow IE (2004). Cyanogenesis in tropical *Prunus turneriana*: characterization variation and response to low light. Funct. Plant Biol.

[b17] Miyazawa Y, Kikuzawa K (2006). Photosynthesis and physiological traits of evergreen broadleaved saplings during winter under different light environments in a temperate forest. Can. J. Bot.

[b18] Niinemets Ü (2010). A review of light interception in plant stands from leaf to canopy in different plant functional types and in species with varying shade tolerance. Ecol. Res.

[b19] Niinemets U, Tenhunen JD (1997). A model separating leaf structural and physiological effects on carbon gain along light gradients for the shade-tolerant species *Acer saccharum*. Plant, Cell Environ.

[b20] Oguchi R, Hikosaka K, Hiura T, Hirose T (2008). Costs and benefits of photosynthetic light acclimation by tree seedlings in response to gap formation. Oecologia.

[b21] Poorter L, Bongers F (2006). Leaf traits are good predictors of plant performance across 53 rain forest species. Ecology.

[b22] Reich PB, Walters MB, Ellsworth DS, Vose JM, Volin JC, Gresham C (1998). Relationships of leaf dark respiration to leaf nitrogen, specific leaf area and leaf life-span: a test across biomes and functional groups. Oecologia.

[b23] Schreiber U, Schliwa U, Bilger W (1986). Continuous recording of photochemical and non-photochemical chlorophyll fluorescence quenching with a new type of modulation fluoremeter. Photosynth. Res.

[b24] Valladares F, Allen MT, Pearcy RW (1997). Photosynthetic responses to dynamic light under field conditions in six tropical rainforest shrubs occuring along a light gradient. Oecologia.

[b25] Walters MB, Reich PB (2000). Trade-offs in low-light CO_2_ exchanges: a component of variation in shade tolerance among cold temperature tree seedlings. Funct. Ecol.

[b26] Whitmore TC (1989). Canopy gaps and the two major groups of forest trees. Ecology.

[b27] Wyka TP, Oleksyn J, Żytkowiak R, Karolewski P, Jagodziński AM, Reich PB (2012). Responses of leaf structure and photosynthetic properties to intra-canopy light gradients: a common garden test with four broadleaf deciduous angiosperm and seven evergreen conifer tree species. Oecologia.

[b28] Xiang S, Reich PB, Sun S, Atkin OK (2013). Contrasting leaf trait scaling relationships in tropical and temperate wet forest species. Funct. Ecol.

[b29] Yoshimura K (2010). Irradiance heterogeneity within crown affects photosynthetic capacity and nitrogen distribution of leaves in *Cedrela sinensis*. Plant, Cell Environ.

